# Protocol for isolating cervico-vaginal fluid cells from *Macaca mulatta* to study immunological and functional changes during pregnancy

**DOI:** 10.1016/j.xpro.2025.103927

**Published:** 2025-07-11

**Authors:** Daniel Short, Monica Cappelletti, Anne Vu, Mark R. Johnson, Suhas G. Kallapur, Pietro Presicce

**Affiliations:** 1 Department of Metabolism, Digestion and Reproduction, Imperial College London, London SW7 2AZ, UK; 2 Department of Pathology and Laboratory Medicine, UCLA Immunogenetics Center, Los Angeles, CA 90095, USA; 3 Divisions of Neonatology and Developmental Biology, David Geffen School of Medicine at the University of California, Los Angeles, Los Angeles, CA 90095, USA

**Keywords:** Cell isolation, Flow Cytometry, mmunology

## Abstract

Cervico-vaginal fluid represents an important biological fluid due to its proximity to gestational tissues, such as the cervix, fetal membranes, and myometrium. Here, we present a protocol to isolate cervico-vaginal fluid cells from *Macaca mulatta*. We describe steps for cervico-vaginal fluid collection, cervico-vaginal cell isolation for nuclear staining and immunophenotyping by flow cytometry, and protein-level detection and quantification by ELISA. This protocol can provide insights into altered immunological changes at the cervico-vaginal interface.

## Highlights

• Instructions for optimal cervico-vaginal lavage collection in pregnant rhesus macaques
• Protocol to characterize cervico-vaginal lavage cells by flow cytometry
• Steps for protein-level detection in cervico-vaginal lavage by ELISA

## Abstract (editor highlights)

Publisher’s note: Undertaking any experimental protocol requires adherence to local institutional guidelines for laboratory safety and ethics.

## Graphical abstract

**Figure:**
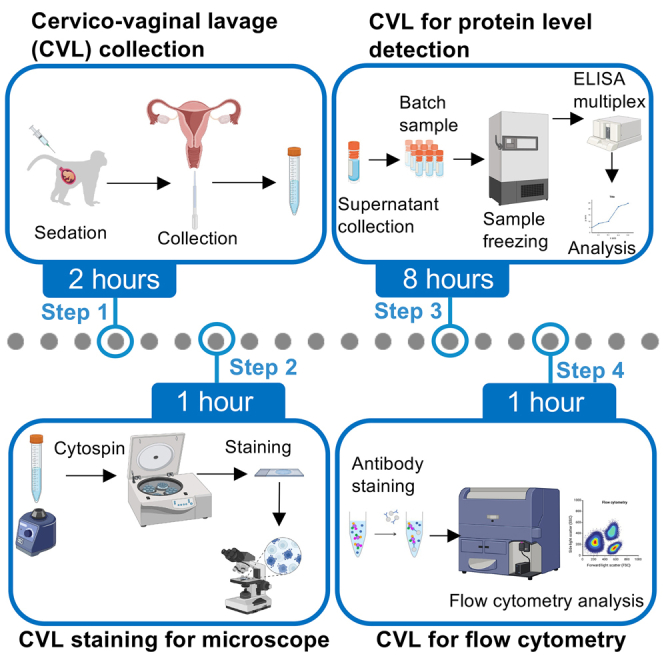

## Before you begin

### Institutional permissions

The protocol below outlines the specific steps for isolating cervico-vaginal lavage cells in non-human primate model of intrauterine infection during pregnancy.^1^ However, this technique may also have potential as a non-invasive method for assessing cervico-vaginal fluid in pregnant women presenting with threatened preterm labor or suspected subclinical chorioamnionitis. All animal procedures must be approved by the Institutional Animal Care and Use Committees (IACUC). Non-human primate cervico-vaginal fluid was collected under the IACUC (protocol # 22121) at the University of California Davis during experimental conditions leading up to preterm birth.

Your institution may require additional and/or region-specific permissions as well as restrictions with some materials/reagents. Therefore, do not start working before these approvals have been obtained.

### Preparation of items for cervico-vaginal lavage collection

**Timing: 30 min**

1. Biological samples from non-human primates (genus *Macaca*) should be considered potentially infected with the endemic Herpes B virus. Additionally, Rhesus macaques used in the current protocol have been exposed to live *E. coli*, making the use of a class II biosafety (BSL2) cabinet highly recommended.
2. Decontaminate a class II biosafety (BSL2) cabinet with UV light.
3. In the BSL2 cabinet, place a container with 10% volume per volume (v/v) bleach solution to collect liquid waste.
4. Place absorbent pads, tube racks, and pre-labeled cryotubes in the BSL2 cabinet.
5. Pre-label two FACS tubes/sample/time point: unstained and full stained.
6. Pre-label one sterile 15 ml tube/time point with animal’s ID number and date of CVL collection.
7. Pre-chill centrifuge to 4°C 15 min prior to collection of the samples.
8. Pre-chill microcentrifuge to 4°C 15 min prior to the collection of the samples.
9. Fill a bucket with enough wet ice to keep the samples and tubes chilled and preserve cell viability.

## Key resources table

**Table:** 

REAGENT or RESOURCE	SOURCE	IDENTIFIER
**Antibodies**		

Live/Dead (human/rhesus) – dilution 1:200	Thermo Fisher Scientific	Cat# L34957
Anti-nonhuman primate CD45 (clone D058-1283) – dilution 1:10	BD Biosciences	Cat# 562394; RRID:AB_756078
Anti-human CD3 (clone SP34-2) – dilution 1:10	BD Biosciences	Cat# 557757; RRID:AB_396863
Anti-human CD56 (clone NCAM16.2) – dilution 1:10	BD Biosciences	Cat# 335809, RRID:AB_399984
Anti-human HLA-DR (clone L243) – dilution 1:10	BioLegend	Cat# 307637, RRID:AB_10895753
Anti-human CD88 (clone MCA2059A647T) – dilution 1:10	Bio-Rad	Cat# MCA2059A647T, RRID:AB_1102420
Anti-human CD19 (clone HIB19) – dilution 1:10	BioLegend	Cat# 302226, RRID:AB_493751
Anti-human CD20 (clone 2H7) – dilution 1:10	BioLegend	Cat# 302322, RRID:AB_493753
Anti-human CD14 (clone M5E2) – dilution 1:10	BioLegend	Cat# 301828, RRID:AB_2275670
Anti-human CD123 (clone 7G3) – dilution 1:10	BD Biosciences	Cat# 558663, RRID:AB_1645485
Stabilizing fixative	BD Biosciences	Cat# 338036

**Biological samples**		

Non-human primate cervico-vaginal lavage	University of California Davis	

**Chemicals, peptides, and recombinant proteins**		

Dulbecco’s phosphate-buffered saline (DPBS) w/o Ca2^+/^Mg2^+^	Thermo Fisher Scientific	Cat# 14190144
Trypan blue stain	Invitrogen	Cat# T1082
Fetal calf serum	Thermo Fisher Scientific	Cat# 16000-044
Human IgG	Sigma	Cat# I2511
0.9% Sodium chloride injection, USP	Pfizer	Cat# 00409-4888-10

**Critical commercial assays**		

Differential Quick Stain Kit	Electron Microscopy Sciences	Cat# 26096-50
Non-human primate cytokine magnetic bead panel – Immunology multiplex assay	MilliporeSigma	Cat# PRCYTOMAG-40K

**Experimental models: Organisms/strains**		

*Macaca mulatta*, females, 6–10 years old	University of California Davis	

**Software and algorithms**		

BD FACSDiva	BD Biosciences	http://www.bdbiosciences.com/us/instruments/research/software/flowcytometry-acquisition/bdfacsdivasoftware/m/111112/features
FlowJo, version 10	FlowJo	https://www.flowjo.com
Belysa	Millipore	https://www.emdmillipore.com

**Other**		

Cytospin	Shandon	Cytospin 3
Funnels for Cytospin	Fisher Scientific	Cat# 22-045-303
Microscope slides	Fisher Scientific	Cat# 12-550-15
Stainless steel Cytoclips	Fisher Scientific	Cat# 59-910-052
Filter cards for Cytospin	Fisher Scientific	Cat# 22-030410
Sharp container	Fisher Scientific	Cat# 14-827-63
96-well U-bottom plate	Corning	Cat# 351177
15 mL conical tubes	Thermo Scientific	Cat# 339650
1.5 mL sterile tubes	Fisherbrand	Cat# 05-408-129
Disposable hemocytometer	Incyto	Cat# DHC-N01-5
Trypan blue stain	Gibco	Cat# 15250-061
Flow cytometry analyzer	BD Biosciences	Fortessa
Centrifuge	Beckman Coulter	Allegra X-I4R
Mini centrifuge	Beckman Coulter	Microfuge 20R
5 mL polystyrene round-bottom tube with cell strainer cap	Corning	Cat# 352235
−80°C freezer	Thermo Fisher Scientific	Cat# TDE40086FA
Syringes	Fisher Scientific	Cat# 05-561-65
Transfer pipettes	Fisher Scientific	Cat# 13-711-9D
Cryotubes	Greiner	Cat# 123279
Luminex 200	Luminex	Cat# APX10031
Absorbent pads	Fisher Scientific	Cat# 22-037-950

## Materials and equipment

**Table:** Flow cytometry wash buffer

Reagent	Final concentration	Amount
1 x PBS		9.8 mL
Heat inactivated fetal calf serum (FCS)	2%	200 μL
**Total**		**10 mL**

Keep on ice. Can be stored for up to 1 week at 4°C.

**Table:** Flow cytometry staining buffer

Reagent	Final concentration	Amount
1 x PBS		9.6 mL
Heat inactivated fetal calf serum (FCS)	2%	200 μL
Human IgG	20 μg/mL	200 μL
**Total**		**10 mL**

Should be made fresh on the day of use and kept on ice.

**Table:** Surface marker master mix (per tube; optimized for 0.5-1×10^6^ cells)

Reagent	Final concentration	Amount
Flow cytometry staining buffer		4.75 μL
Live/Dead Fixable Aqua Dead Cell Stain Kit	1:200	0.25 μL
Anti-CD45	1:10	5 μL
Anti-CD88	1:10	5 μL
Anti-HLA-DR	1:10	5 μL
Anti-CD14	1:10	5 μL
Anti-CD19	1:10	5 μL
Anti-CD20	1:10	5 μL
Anti-CD3	1:10	5 μL
Anti-CD56	1:10	5 μL
Anti-CD123	1:10	5 μL
**Total**		**50 μL**

Should be made fresh on the day of use and kept at 4°C protected from light with foil.

## Step-by-step method details

### Cervico-vaginal lavage collection

**Timing: 2 h**

**Figure 1:**
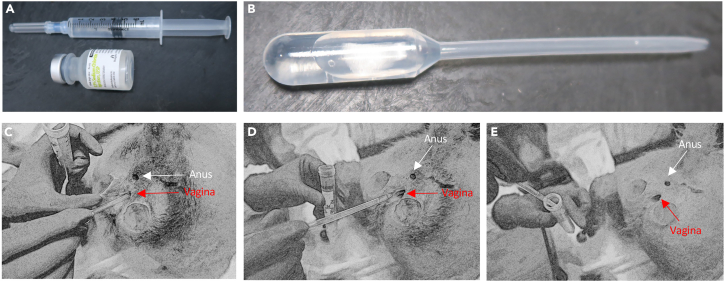
CVL collection from non-human primates (A) Fill a sterile 5 mL syringe with 4 ml sterile saline solution. (B) Transfer 4 ml of sterile saline solution from the syringe to a sterile transfer pipet. (C) Insert the pipet in the vagina of the sedated animal and flush out the saline solution which will mix with the cervico-vaginal fluid. (D) Suction the mixture of cervico-vaginal fluid and saline solution. (E) Transfer the aspirated cervico-vaginal fluid to a sterile 15 ml tube and store on ice immediately.

In this section, we describe in detail how to sedate and position the non-human primate (Rhesus macaque) and collect cervico-vaginal lavage (CVL) (Figure 1). Figure 1 shows the procedure for non-human primates. All animals should be correctly identified using departmental codes and/or numbers and sedated in line with the departmental policies. With the right approval and without the need of sedation, the cervico-vaginal lavage can also be collected from pregnant women. Safe handling and disposal of sharps is essential. Never recap a needle and use a sharp container for disposal.

1. Ensure the correct animal has been identified by checking for the identification number.

***Note:*** Squeeze mechanisms can be used to restrain animals housed indoors, where nets and appropriate additional personal protective equipment will be required for outdoor animals.

2. Standard dosing when using ketamine as a tranquilizer for Rhesus macaques is 10 mg/kg. There may be institutional variations for dosing, and for other species of non-human primate.
3. The most recent weight taken from the animal’s care record should be used. The most recent weight should be measured at the beginning of an experiment cycle.
4. Aspirate the ketamine into the syringe.
5. Administration is by intra-muscular injection into a large muscle group, preferably the anterior quadriceps.
6. Ensure the needle has not been inserted into a blood vessel by aspirating the syringe (see the troubleshooting section – problem 1).
7. After you have confirmed you are within a muscle group and not a blood vessel, inject the ketamine.
8. With correct dosing the following timelines apply:
  a. 3–5 min for complete immobilization to occur.
  b. 40 min of sedation.
  c. 20–40 min for a typical recovery time.

**CRITICAL:** It is important to keep the animal sedated for a short a time as possible. CVL must be collected within 15 min after animal sedation and immediately stored on ice. Length of sedation however should not affect the contents of the CVL.

9. Once you have confirmed the animal is fully sedated and immobilizes as in step 8b, proceed to release the animal from any restraints:
  a. For indoor animals, release the squeeze mechanism of the cage.
  b. For outdoor animals, remove any manual restraints and hold the animal to ensure it remains under close observation and cannot injure itself until it is immobilized.

***Note:*** Animals should always be observed closely as the medication takes effect. Care should be taken to ensure the animal does come to harm or assume a compromised position during this time.

10. Once the animal is immobilized it can be removed from the cage.

***Note:*** Continuous monitoring for evidence of movement of limbs or changes to respiratory patterns is essential during all procedures performed under sedation (see the Troubleshooting section – Problem 2).

11. Fill a syringe with 4 mL of sodium chloride solution (i.e., sterile saline solution) (Figure 1A).
12. Transfer the sterile saline solution from the syringe to a sterile transfer pipet (Figure 1B).
13. When the animal is appropriately sedated, place the animal in a prone position with its rump elevated.
14. The perineum should be cleansed with saline and gauze.
15. The transfer pipette should be inserted carefully into the vagina up to the level of the cervix (4–6 cm). Take care not to damage the vaginal or perineal tissues (Figure 1C).
16. Slowly flush the sterile fluid into the vagina and aspirate back using the transfer pipette (Figure 1D).
17. Transfer the retrieved cervico-vaginal lavage (aspirated back from the vagina in Step 16) into a pre-labeled 15 ml sterile conical tube (Figure 1E).
18. Place this 15 mL conical tube containing the CVL on ice.
19. Document all procedures in the animal’s medical record.
20. After CVL collection, follow:
  a. Steps 21–34 for cervico-vaginal immune/non immune cell isolation, cell count, and staining to investigate cell composition by microscopy.
  b. Steps 35–41 for protein level detection and analysis.
  c. Steps 42–62 for flow cytometry staining and analysis.

### CVL cell staining for microscopy

**Timing: 1 h**

In this section, we describe in detail the steps for optimal CVL cell staining. We recommend beginning with fresh CVL.

**Figure 2:**
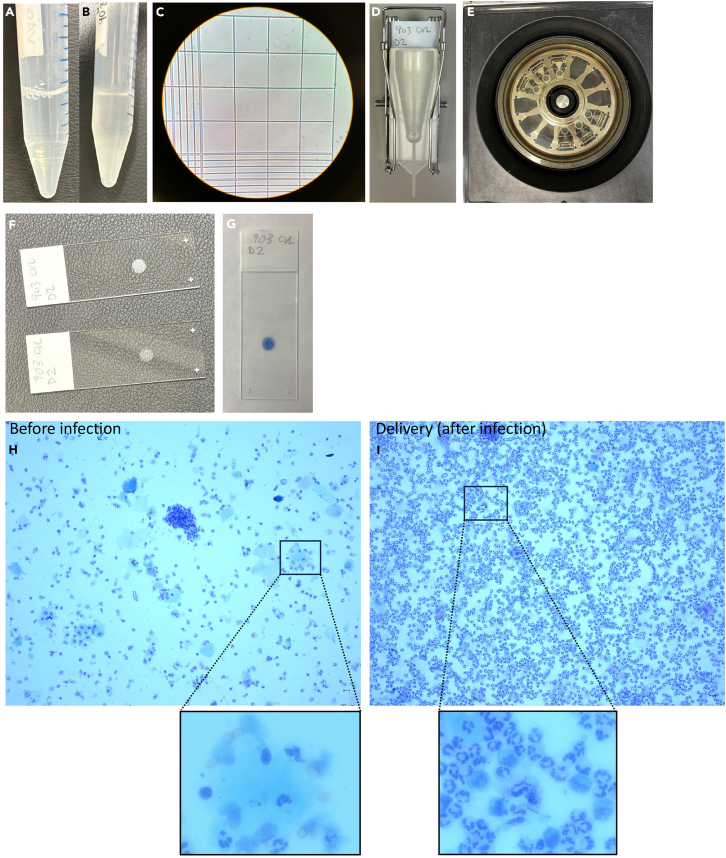
CVL cell staining for microscopy CVL appearance can vary according to the gestational age, the presence of inflammation and/or the presence of labor. (A) CVL collected before infecting the animal with *E. coli.* Note that the CVL is clear. (B) CVL collected at delivery and after six days of *E. coli* exposure. Note that the CVL is cloudy. (C) Resuspend the CVL and perform cell count with trypan blue exclusion test. Record cell count and viability. (D) Label two microscope slides for each sample and mount them with a stainless steel Cytoclip, filter card, and a disposable Cytofunnel sample chamber. (E) Place the mounted slides in the Cytospin. Dispense 125 μL (0.5x10ˆ6 cells)/chamber in the Cytospin. Spin the slides for 5 min at 1600 rpm. (F) After centrifugation, disassemble chambers carefully. The cells are visible on the slides. (G) Stain the slides with Differential Quick Stain Kit. (H) Representative microscope images showing CVL before injecting the animal with *E. coli* in the amniotic fluid (∼140 ga). Note the low number of neutrophils. Bars = 20 μm. (I) Representative microscope images showing CVF at delivery (∼143 ga, i.e., after ∼3 days of *E. coli* exposure). Note the massive neutrophil infiltration. Bars = 20 μm.
22. Record the volume of CVL.
23. Vortex the conical tube containing CVL to ensure there is no cell pellet at the bottom.
  a. In a 96-well U-bottom plate, dilute the CVL 1:1 with trypan blue if the CVL appears clear, as shown in Figure 2A.
  b. If the CVL appears cloudy, as shown in Figure 2B, dilute it 1:5 with trypan blue.
  c. Resuspend the CVL/trypan blue mixture, load 10 μL into the disposable hemocytometer.
  d. Perform the cell count.
24. Record cell count and viability (Figure 2C). Cell viability is usually >99% (confirmed also by flow cytometry as shown in Figure 4D). However, dead cells should not be removed. The ideal number of cells/volume to load onto each microscope slide is 0.5 × 10^6^/125 μL (see the troubleshooting section – problem 3).
25. Adjust the cell concentration at 1 × 10^6^/250 μL with 1X PBS (see the troubleshooting section – problem 4).
26. Label two microscope slides/each sample/each time point – see key resources table.
27. Mount the sample holder following this order (Figure 2D):
  a. Stainless steel Cytoclip.
  b. Pre-labeled microscope slide.
  c. Filter card.
  d. Disposable Cytofunnel sample chamber.
28. Place the chambers in the Cytospin (Figure 2E).
29. Dispense 125 μL/chamber.
30. Spin the chambers for 5 min at 1600 rpm.
31. Carefully remove the microscope slides from the holders and let them dry for 10 min at room temperature (Figure 2F).
32. Stain the microscope slides with Differential Quick Stain Kit.
33. Let the microscope slides dry for 15 min at room temperature (Figure 2G).
34. Investigate the cell composition at different stage of pregnancy and before/after the infection under the microscope (Figures 2H and 2I).

***Note:*** Cell composition varies significantly during different stages of pregnancy and in the presence of infection. For example, epithelial cells predominantly constitute the cell population in the absence of infection. However, upon infection, the number of neutrophils increases substantially.^2^

21. CVL may appear clear (Figure 2A) or cloudy (Figure 2B) based on the presence of infection/inflammation and/or the proximity of labor and depending on the gestational age.

### CVL for protein-level detection

**Timing: 8 h**

In this section, we describe in detail the steps for optimal CVL supernatant collection for detection of protein levels. We recommend beginning with fresh CVL.

**Figure 3:**
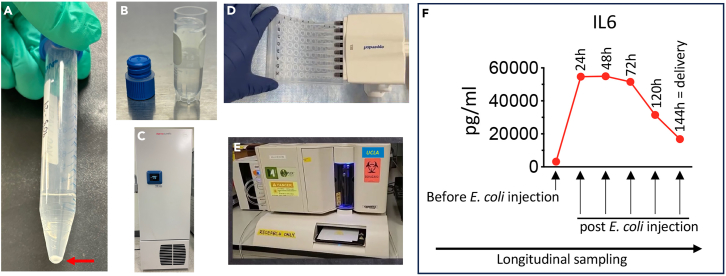
Protein-level detection in CVL (A) After cell counting, pellet the CVL (7 min at 600 g, at 4°C). Red arrow shows the CVL cell pellet upon centrifugation. (B) Without disturbing the pellet, transfer ∼1 ml of into a sterile cryotube. (C) Store the samples at −80°C. (D) Add the samples in the ELISA multiplex plate. (E) Load the plate into the Luminex 200. (F) Example of data analysis. CVL was collected longitudinally and IL6 protein concentration was graphed.

35. Pellet the leftover CVL from step 25 in the same 15 ml tube for 7 min at 600 x g (4°C) (Figure 3A).

**Figure 4:**
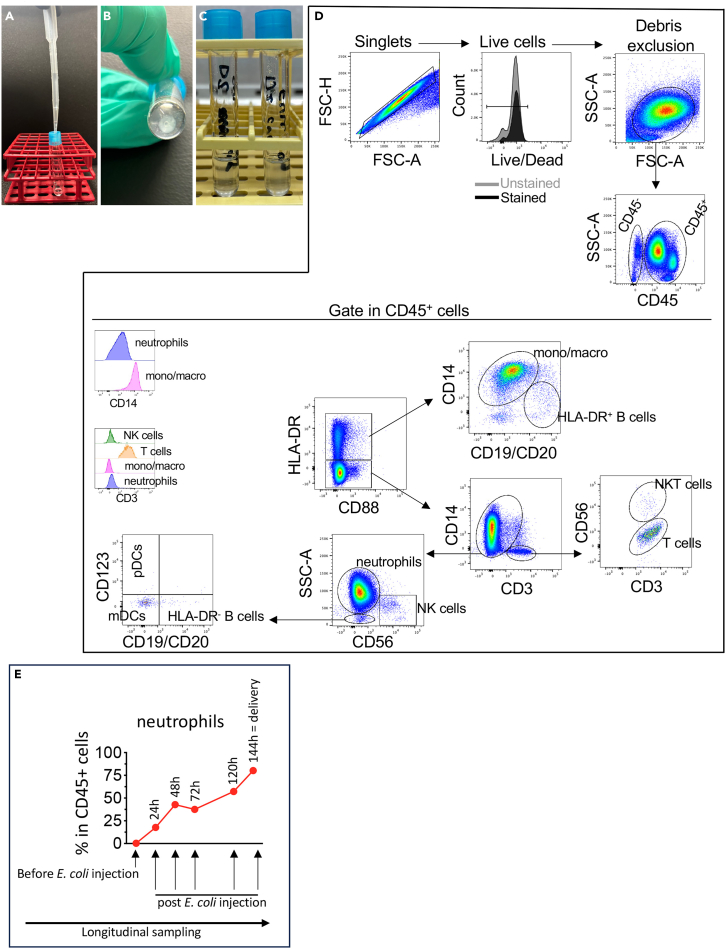
Gating strategy for flow cytometry phenotype analysis of CVL cells (A) Filter the CVL cell solution in a FACS 5 ml polystyrene round-bottom tube with cell-strainer cap. (B) After centrifugation, the cells pellet is visible. (C) After incubating the cells with the Ab mix, add 500 μl/tube of cold FACS washing buffer. (D) Representative density plots of Rhesus CVL samples. Forward scatter area (FSC-A) and forward scatter height (FSC-H) gating on cells to exclude doublets. Dead cells were excluded by Live/Dead Fixable Aqua Dead Cell Stain kit. FSC and side (SSC) scatter gating on cells to exclude cellular debris. CD45^−^ cells gate representing non-leukocyte cells and CD45^+^ cells positive gating representative leukocyte cells. Inside the CD45^+^ cells, the leukocyte subpopulations were gated as monocytes/macrophages (CD3^−^HLA-DR^+^CD19^−^CD20^−^CD14^+^); activated B cells (CD3^−^HLA-DR^+^CD14^−^CD19^+^CD20^+^); neutrophils (CD3^−^CD14^low^HLA-DR^−^CD88^+^CD56^−^); NK cells (CD3^−^CD14^−^HLA-DR^−^CD56^+^); resting B cells (CD3^−^HLA-DR^−^CD14^−^CD56^−^CD19^+^CD20^+^); T cells (CD14^−^CD56^−^CD3^+^); and NKT cells (CD14^−^CD3^+^CD56^+^); myeloid dendritic cells (mDCs, CD3^−^CD14^−^HLA-DR^−^CD56^−^CD19^−^CD20^−^CD123^−^); plasmacytoid dendritic cells (pDCs, CD3^−^CD14^−^HLA-DR^−^CD56^−^CD19^−^CD20^−^CD123^+^). A similar gating strategy can be used to characterize human cells, with the only difference that CD66b should be used instead of CD88 for neutrophils. (E) Example of data analysis: frequency of neutrophils in CVL at different time points during gestation (before *E. coli* injection = gestational age 140 days; delivery = gestational age gestational age 146 days).
36. Save the supernatant in a pre-labeled cryotube without disturbing the pellet (Figure 3B).
37. Repeat Steps 1–36 to collect all the samples needed according to the experimental plan (such as different time points, different treatment, etc.) and store them at −80°C (0.5–1 ml/each) (Figure 3C).
38. Thaw all the samples on ice at the same time to avoid multiple cycles of thawing/freezing.
39. Load the samples into the ELISA plate (Figure 3D) (see the troubleshooting section – problem 5) (https://www.sigmaaldrich.com/deepweb/assets/sigmaaldrich/product/documents/172/113/protocol-prcytomag40k-ms.pdf).
40. Load the plate into the ELISA reader Luminex 200 (Figure 3E).

**CRITICAL:** Times varies according to the kit used to detect cytokines as well as the machine used. The Luminex 200 system used in this protocol for higher sample throughput typically reads a 96-well plate in 45 min.

41. Example of data analysis: IL-6 protein levels in CVL at different time points during gestation (Figure 3F).

### CVL for flow cytometry

**Timing: 1 h**

In this section, we describe in detail the steps for optimal CVL cell staining for flow cytometry. We recommend beginning with fresh CVL.

42. Prepare the antibody mix as indicated in the key resources table and keep it at 4°C covered in foil to protect from light. A custom mix can be prepared based on the experimental requirements and the machine being used.
43. Pre-label two FACS 5 ml polystyrene round-bottom tubes (unstained and stained).
44. After collecting the supernatant as described in step 37, resuspend the cell pellet depicted in Figure 3A in 1 ml of PBS (see the troubleshooting section – problem 6).
45. In a FACS 5 ml polystyrene round-bottom tube with cell-strainer cap, add the 1 ml CVL cell solution of step 46 to eliminate cell aggregates (Figure 4A).
46. Replace the cell-strainer cap with a new one. Spin the tube with the CVL solution w/o cell aggregates for 4 min at 930 x g at 4°C (Figure 4B).
47. Discard the supernatant without disturbing the cell pellet.
48. Resuspend the cells in 1 ml of PBS and count the cells as described in step 23.
49. Spin the tube for 4 min at 930 x g at 4°C.
50. Discard the supernatant without disturbing the cell pellet.
51. Resuspend 0.5–1 × 10^6^ cells in 50 μL of blocking buffer.
52. Dispense 50 μL of cell solution/tube in the pre-labeled tubes “unstained” and “full stained” (see step 5 in the section “Preparation of items for cervico-vaginal (CVL) collection”).
53. Incubate for 10 min at 4°C covered in foil to protect from light.
54. Add the antibody cocktail in the “full stained” tube and 50 μL of FACS buffer in the “unstained” tube.
55. Cover the tubes in foil to protect from the light and incubate for 20 min at 4°C.
56. In the tube(s) of step 54, add 500 μL/tube of cold FACS washing buffer (Figure 4C).
57. Spin the tubes for 4 min at 930 x g at 4°C.
58. Carefully aspirate the supernatant without disturbing the cell pellet.
59. After removing the supernatant as indicated in step 58, repeat steps 56→58.
60. Resuspend the cell pellet in 200 μL of BD stabilizing solution.
61. It is recommended to acquire at least 500,000 event/tube of live cells (see the troubleshooting section – problems 7 and 8). Representative flow cytometry gating strategy is shown in Figure 4D. The surface marker master mix used in this protocol allowed to characterize different immune cells such as macrophages, B cells, T cells, NKT cells, NK cells, neutrophils, plasmacytoid and myeloid dendritic cells.
62. Example of data analysis: frequency of neutrophils in CVL at different time points during gestation (Figure 4E).

***Note:*** The frequencies of CD45^−^ non-immune cells and CD45^+^ immune cells, along with the various CD45^+^ immune cell populations, change significantly during different stages of pregnancy and in response to infection.^3^

***Note:*** After each centrifugation, carefully aspirate the supernatant because cell pellet may be loose.

## Expected outcomes

The protocol is optimized for the isolation of cervico-vaginal lavage (CVL) cells for cell staining for microscope analysis, phenotypic characterization by flow cytometry, and protein detection by ELISA multiplex. Approximately, 1 ml of cervico-vaginal lavage will result in 0.1–9 × 10^6^ cells. The number of isolated cells depends greatly on the gestational age, the presence of infection/inflammation and/or the onset of labor as shown in Figures 2H and 2I.

## Limitations

The cervico-vaginal lavage should be kept on ice and the protocol works best when using fresh lavage. Cervico-vaginal lavage samples up to 48 h after collection can be used, but cell viability may decrease. For protein level detection, freeze the supernatant as soon as possible at −80°C and avoid freeze-thawing cycles.

## Troubleshooting

### Problem 1

The needle aspirates blood (related to Step 6).

### Potential solution

Carefully remove the needle and repeat step 5.

### Problem 2

The animal is not sedated enough (related to step 10).

### Potential solution

Additional sedative should be administered.

### Problem 3

The cell count is too low (related to step 24).

### Potential solution

Instead of loading 0.5 × 10^6^ cells/slide, decrease the number of cells. The low number of cells may not be caused by an issue with the sample, but it could really reflect a particular stage of pregnancy, the absence of the inflammation, and/or the absence of labor (see also Figure S1).

### Problem 4

The sample is too diluted (related to step 25).

### Potential solution

Pellet the cells in a 1.5 ml tube and resuspend them in PBS at a concentration of 1 × 10^6^ cells/250 μL.

### Problem 5

The CVL is viscous and therefore resuspending the samples is extremely difficult (related to step 39).

### Potential solution

Dilute the sample with the dilution buffer provided by the kit (See key resources table) and record the dilution factor to calculate the final concentration of the proteins.

### Problem 6

The pellet is bloody (related to step 44).

### Potential solution

• Add 10 ml of ACK lysis buffer, incubate at RT for 7 min.
• Fill the tube with sterile PBS, pellet the cell solution for 7 min at 600 x g (RT);
• Aspirate and discard the supernatant;
• Wash with PBS and pellet the cell solution for 7 min at 600 x g (RT);
• Gently discard the supernatant.

### Problem 7

The flow cytometer is clogged because of epithelial cell aggregates (related to step 61).

### Potential solution

Filter sample through a new FACS 5 ml polystyrene round-bottom tube with cell-strainer cap and re-acquire it.

### Problem 8

Very few events are recorded by flow cytometry (related to step 61).

### Potential solution

The low number of events maybe not caused by an issue with the sample, but it could really reflect a particular stage of pregnancy, the absence of the inflammation, and/or the onset of labor. When pregnancy progresses and/or inflammation and/or labor occur, the number of events increases (Figure S2).

## Resource availability

### Lead contact

Further information and requests for resources and reagents should be directed to and will be fulfilled by the lead contact, Pietro Presicce (ppresicce@mednet.ucla.edu).

### Technical contact

Questions about the technical specifics of performing the protocol should be directed to and will be fulfilled by the technical contact, Daniel Short (d.short22@imperial.ac.uk).

### Materials availability

This study did not generate new unique reagents.

### Data and code availability

This study did not generate or analyze datasets.

## Acknowledgments

We would like to thank Dr. Laura M. Garzel, Diana Diaz, and Paul-Michael Sosa, the research personnel at the California National Primate Center, UC Davis, for their help with the animals. We also thank the Immune Assessment Core (IAC) for the ELISA multiplex. This study was supported by R01 HD98389 (S.G.K.) and by the Core Technology Award from the Immunology, Inflammation, Infection, and Transplantation (I3T) Research theme, David Geffen School of Medicine (DGSOM) at UCLA (P.P.). The graphical abstract was created with BioRender.com.

## Author contributions

D.S., M.C., A.V., and P.P. participated in data generation. D.S., M.C., A.V., M.R.J., S.G.K., and P.P. participated in the analysis and interpretation of the data. S.G.K. and P.P. participated in the conception and design of the study, and S.G.K. and P.P. obtained the funding. D.S., M.C., and P.P. wrote the manuscript. All authors have reviewed the manuscript and approved the final version.

## Declaration of interests

The authors declare no competing interests.
