# Supplementary material for: Protocol for isolating cervico-vaginal fluid cells from Macaca mulatta to study immunological and functional changes during pregnancy
Source: STAR Protoc. 2025 Jul 11;6(3):103927. doi: 10.1016/j.xpro.2025.103927 (PMC12274921; doi:10.1016/j.xpro.2025.103927)
Supplement: Document S1. Figures S1 and S2 [file mmc1.pdf]

Supplementary Figure 1.

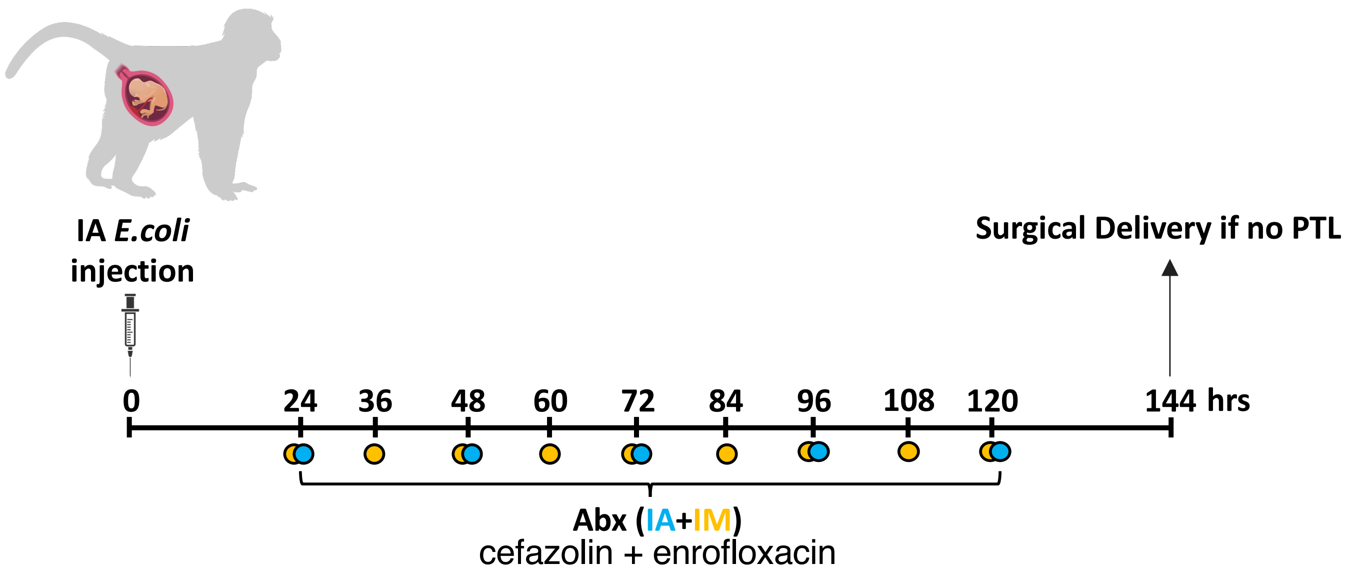

Supplementary Figure 1. Animal Model reflecting the different stages of pregnancy and the presence of infection, Related to step 24. Pregnant Rhesus macaques (*Macaca mulatta*) at ~135d gestational age (~85% term gestation) were given: IA live *E. coli* (10^6 CFU) followed one day later by Antibiotics (Abx, Intramuscular (IM) cefazolin 25 mg/k twice daily + IM enrofloxacin 5mg/k twice daily. Additionally, IA injection of cefazolin (10mg) + IA enrofloxacin (1mg) were given once daily). Cervico-vaginal lavage (CVL) samples were collected daily. (PTL=preterm labor)

Supplementary Figure 2.

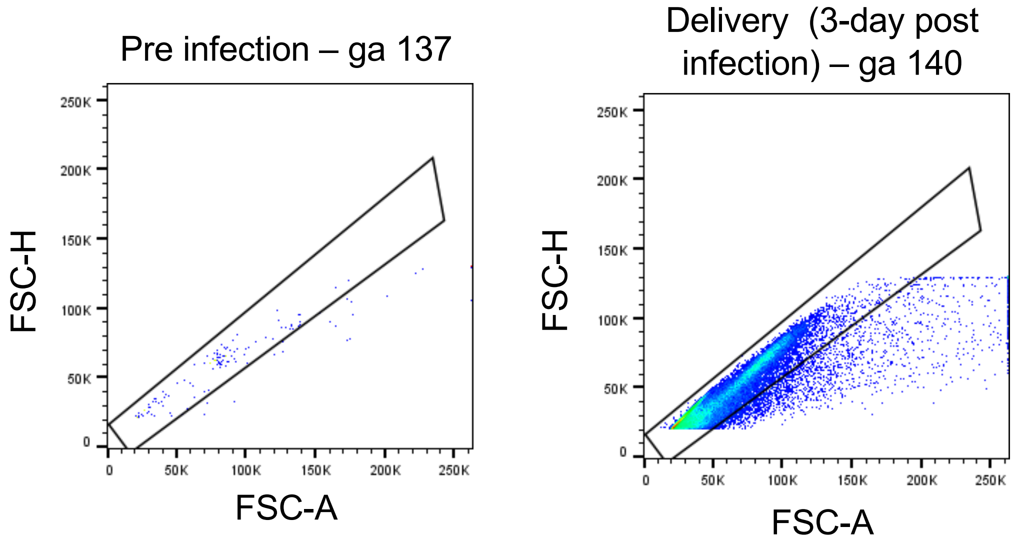

Supplementary Figure 2. Numbers of events change dramatically, Related to Step 61. Left: Few events may reflect a particular stage of pregnancy and/or the absence of the inflammation and/or the onset of labor. Right: The numbers of events increase dramatically after infection and/or with the gestation progress (ga=gestational age).
